# Alisol A Alleviates Arterial Plaque by Activating AMPK/SIRT1 Signaling Pathway in apoE-Deficient Mice

**DOI:** 10.3389/fphar.2020.580073

**Published:** 2020-11-02

**Authors:** Ke Wang, Beibei Zhang, Dingzhong Song, Jianqiang Xi, Wusi Hao, Jie Yuan, Chenyu Gao, Zhongbao Cui, Zhihong Cheng

**Affiliations:** China State Institute of Pharmaceutical Industry, National Pharmaceutical Engineering and Research Center, Shanghai, China

**Keywords:** Alisol A, AMPK/SIRT1 pathway, anti-atherogenesis, anti-inflammation, PPARα/δ

## Abstract

*A* *lismatis Rhizoma* (zexie), an herb used in traditional Chinese medicine, exhibits hypolipemic, anti-inflammation and anti-atherosclerotic activities. Alisol A is one of the main active ingredients in *Alismatis Rhizoma* extract. In this study, we investigate the role of alisol A in anti-atherosclerosis (AS). Our study demonstrated that alisol A can effectively inhibit the formation of arterial plaques and blocked the progression of AS in *ApoE^−/−^* mice fed with high-fat diet and significantly reduced the expression of inflammatory cytokins in aorta, including ICAM-1, IL-6, and MMP-9. In addition, we found that alisol A increased the expression of PPARα and PPARδ proteins in HepG2 cells and in liver tissue from *ApoE^−/−^* mice. Alisol A activated the AMPK/SIRT1 signaling pathway and NF-κB inhibitor IκBα in HepG2 cells. Our results suggested that alisol A is a multi-targeted agent that exerts anti-atherosclerotic action by regulating lipid metabolism and inhibiting inflammatory cytokine production. Therefore, alisol could be a promising lead compound to develop drugs for the treatment of AS.

## Introduction

Cardiovascular disease is a big threat of human health worldwide. Atherosclerosis (AS) is the pathological basis of coronary heart disease, myocardial infarction and ischemic stroke. It is characterized by the deposition of atheromatous plaques on the walls of blood vessels, leading to arterial stenosis (Lusis, 2000). With the change of diet and the advent of aging society, the incidence of AS is continuously increasing. It is estimated to be the underlying cause of nearly 44% of death in Western countries (Herrington et al., 2016). At present, statins are the major drugs used for the treatment of AS. Statins reduce liver cholesterol synthesis by inhibiting HMG-CoA reductase and decrease low-density lipoprotein cholesterol (LDL-C) levels in plasma (Ray et al., 2014). However, AS is a complex disease caused by the combination of a variety of factors, including excessive activation of macrophages, increased endothelial permeability, and chronic inflammation. Although the lipid-lowering effect of statins has been clinically proven, it was found that statins reduce the incidence of atherosclerotic cardiovascular disease mainly by lowering LDL, and the reduction is only in one-third of patients (Tall, 2017). Besides, satins cause some adverse effects such as myopathy, elevated transaminase, and increased risk of diabetes. Furthermore, statin-associated rhabdomyolysis can be fatal in severe cases (Ward et al., 2019). Therefore, it is an unmet need to find new anti-atherosclerotic drugs.

The traditional Chinese medicine *Alismatis Rhizoma* (zexie) is the dry tuber of *Alisma orientale* (Sam.) Juzep. The 2015 edition of the Chinese Pharmacopoeia indicates that *Alismatis Rhizoma* (AR) has the effect of clearing dampness, promoting diuresis, dispersing heat, reducing turbidity and lipid (Chinese Pharmacopoeia Commission, 2015 Edition). It was found in the early 1960 that AR extract, which contains protostane type triterpenoids, including alisol A, B, C, and their acetates, has the effect of anti-fatty liver (Tadayuki, 1960). It was also shown that AR extract has hypolipemic, anti-diabetic, anti-inflammation, and anti-atherosclerotic activities (Xiyan, 1981; Lau et al., 2008; Han et al., 2013; Zhou et al., 2019). However, the mechanisms of these activities are largely not understood. The peroxisome proliferator-activated receptors (PPARs), particularly PPARα and PPARδ can regulate blood lipids, inhibit inflammatory proliferation and migration of vascular smooth muscle cells, and therefore exhibit anti-atherosclerotic effect (Soskić Sanja et al., 2011; Ajith and Jayakumar, 2016). The 5’-adenosine monophosphate-activated protein kinase (AMPK) has been reported as an important regulator of cell metabolism (Fisslthaler and Fleming, 2009). It exerts anti-atherosclerotic action through reducing the activation of NF-κB (Li et al., 2020), promoting phosphorylation of acetyl-CoA carboxylase (ACC) to inhibit liver lipid synthesis (Ho et al., 2019), and inhibiting mTOR to enhance autophagy of macrophages (Yang et al., 2018). In addition, activation of AMPK increases intracellular nicotinamide adenine dinucleotide (NAD+) levels and activates Sirtuin 1(SIRT1) to protect the heart (Cantó et al., 2009).

Based on our previous finding that alisol A has hypolipidemic effect, we evaluated the therapeutic efficiency of alisol A in an AS mouse model using commercially available *ApoE^−/−^* mice fed with high-fat diet. The mechanisms of action of alisol A was investigated in cultured cell model. The results demonstrated that administration of alisol A can alleviate the established arterial plaque. Furthermore, PPARα/δ, NF-kappa-B inhibitor alpha(IκBα), and AMPK/SIRT1 pathways contribute to the anti-atherosclerotic effect of alisol A.

## Materials and Methods

### Reagents

Alisol A (purity >99%) was prepared in our laboratory. Atorvastatin (purity >98%) was purchased from Pfizer Pharmaceutical. All the other reagents used were of analytical grade.

### Animals

The 6-week-old *ApoE^−/−^* mice of C57BL/6 were purchased from Beijing Huafukang Biotechnology and housed under standard conditions with a 12-h light/dark cycle, temperature at 22 ± 3°C, free access to food and water. Experimental protocols were approved by the China State Institute of Pharmaceutical Industry Animal Management and Ethics Committee. After one week of adaptive feeding of regular feed, they were given high-fat diet (D12492, Hua Fu Kang, Beijing, China) with 60% of kcals from fat for 15 weeks to establish AS model. Ten mice were randomly selected to determine the plaque formation by intravascular high-resolution ultrasonography. Mice were then randomly divided into four groups (n = 15): the AS group (AS), the AS group with atorvastatin (AS+AT; 10 mg/kg/day), the AS group with low-dose of alisol A (AS+A Low; 25 mg/kg/day) and the AS group with high-dose of alisol A (AS+A High; 100 mg/kg/day). Alisol A was intragastrically administrated for 12 weeks, twice a day. Atorvastatin was intragastrically administrated for 12 weeks, once a day. The food intake of these mice was recorded every 3 days, and the body weight of the mice was monitored once every week. At the end of the experiments, mouse blood was collected and centrifuged for 5 min (5000 rmp) to get serum samples. After taking the blood, the mice were sacrificed for collection of liver and aorta tissues.

### Aorta Ultrasound

After 4, 8, and 12 weeks of the experiments, the high-resolution small animal ultrasound system was used to monitor the area and diameter of the aorta plaques in each mouse. *ApoE^−/−^* mice were anesthetized with a mixed gas of 1%–3% isoflurane and pure oxygen, placed flatly on a heated platform (37°C). After applied ultrasound coupling agent to depilated forechest of mice, the high-frequency (18–38 MHz) probe of Vevo^®^ 2100 imaging system (VisualSonics MS400, CA) was used to acquire intravascular images of innominate arteries, to record vascular plaque area and inner diameter, and to quantitatively analyze the progress of atherosclerotic plaque in each mouse.

### Serum Biochemistry Assays

Serum concentrations of triglycerides (TG), total cholesterol (TC), low-density lipoprotein cholesterol (LDL-C), and high-density lipoprotein cholesterol (HDL-C) were determined by automatic biochemical analyzer Chemray 240 (Rayto, shenzhen, China).

### H&E, Oil Red O, and Immunohistochemical Staining on Aortic Samples

Aorta was fixed with 4% PFA, embedded in paraffin, and cut into 5‐μm‐thick sections using a microtome. H&E staining was performed according to standard protocol. For Oil Red O staining, aorta was sectioned longitudinally, stained in Oil Red O for 2 h, and then immersed in 70% ethanol until the plaques of the specimen become red and the non-plaque blocks appear white. For immunohistochemistry analysis, sections were incubated with primary antibody (Abcam, Cambridge, UK,1:100) diluted in blocking solution overnight at 4°C. Following incubation with HRP-conjugated secondary antibody, the sections were counterstained with hematoxylin and developed with diaminobenzidine.

### Cell Culture and Drug Treatment

Human hepatocellular carcinoma cell (HepG2) was purchased from the Tissue Culture Collection of the Chinese Academy of Sciences, Shanghai, China. Cells were cultured in Dulbecco’s modified Eagle’s medium (DMEM) supplemented with 10% of fetal bovine serum (FBS) at 37°C and 5% CO_2_ atmosphere. After the cells grew to about 80% confluent, free fatty acid (FFA) (OA: PA = 2: 1, 1mM) and alisol A (1, 5, 10, 20 μM) were added and incubated for 24 h.

### Assessment of Cytotoxicity

Cells (5 × 10^3^ per well) were seeded into a 96-well tissue culture plate. After overnight culture, indicated concentrations of alisol A (0, 1, 5, 10, 20, 30, 40, 60, 80, and 100 μM) were added. Cell viability was assessed by CCK-8 assay (Beyotime Biotechnology, China) according to the manufacturer’s protocols. The absorbance at 450 nm was measured by using a microplate reader (Bio-Rad, USA).

### Oil Red O Staining

The cells fixed with 4% PFA were washed three times with cold PBS and stained for 30 min at 37°C with the Oil Red O working solution. Images of the positively stained cells (red) were acquired by a light microscope (Nikon TS100 inverted microscope).

### RNA Purification and Quantitative Real-Time PCR

The total RNA was isolated using TRIzol reagent (Sangon Biotech, China). RT‐qPCR was performed with the StepOne fluorescence quantitative PCR instrument (ABI, Foster, CA, USA) according to the manufacturer’s instructions, β-actin was used as an internal control. The primers used in this study were in the **Table 1**.

**Table 1:** Specific primers of this study.

Gene names	primers
*β-actin*	forward, 5’-TAGTTGCGTTACACCCTTTCTTG-3’ reverse, 5’-TCACCTTCACCGTTCCAGTTT-3’
*FAS*	forward, 5’-GGCTCCACCAAGTCCAACAT-3’ reverse, 5’-GGGCTATGGAAGTGCAGGTT-3’
*SIRT1*	forward, 5’-GGTCAAGGGATGGTATTTATGC-3’ reverse, 5’-CAGCGTGTCTATGTTCTGGGTA-3’
*IκBα*	forward, 5’-TCCATCCTGAAGGCTACCAA-3’
	reverse, 5’-CCCAAGGACACCAAAAGCT-3’

The gene primers are provided by Sangon Biotech company.

### Western Blotting

Tissues and Cultured cells were lysed with RIPA buffer supplied with phosphatase and protease inhibitor cocktail (Beyotime Biotechnology, China). Concentrations of protein were determined by the bicinchoninic acid (BCA) assay (Beyotime Biotechnology, China). Total protein extract (40 μg) was separated by 10% SDS polyacrylamide gel electrophoresis and transferred to PVDF membranes (BioRad, America). The primary antibodies were as follows: anti-PPARα, anti-PPARδ, anti-AMPK, anti-p-AMPK, anti-SIRT1, and anti-IκBα (1:1,000; Cell Signalling Technology, Abcam). Immunoreactive bands were quantitatively analyzed using ImageJ software.

### Statistical Analysis

The results were expressed as the mean ± standard deviation (SD). Comparisons between two groups were carried out using two-tailed unpaired Student’s t-test. The one‐way ANOVA with the NewmaneKeus multiple comparison test was performed to determine the significance of the differences between groups. The statistical analyses were performed with Graphpad Prism 5, and *p* < 0.05 were regarded as significant.

## Results

### Alisol A Attenuated High-Fat Diet–Induced Body Weight Gain

**Figure 1:**
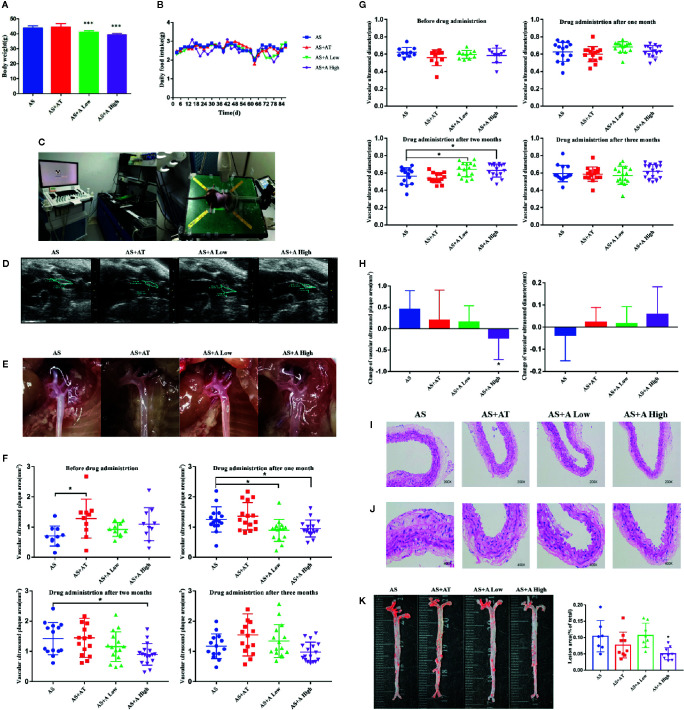
Alisol A reduces the body weight of ApoE*^−/−^* mice without interfering the normal diet and high dose of alisol A promotes plaque regression. **(A)** Body weights of the four groups of mice, including the AS group (atherosclerosis group), the AS+AT group (atherosclerosis group with atorvastatin), the AS+A low group (atherosclerosis group with low-dose of alisol A) and the AS+A high group (atherosclerosis group with high-dose of alisol A), n=13-15. **(B)** Food intake of ApoE*^−/−^* mice. **(C)** High-resolution small animal ultrasound instrument. **(D)** High-resolution small animal ultrasound system diagram. **(E)** Representative images of vascular aorta. **(F)** Change of aortic arch ultrasound plaque area in 1-3 months after administration. **(G)** Change of vascular ultrasound diameter in 1-3 months after administration. **(H)** Change of vascular ultrasound plaque area and diameter in each group after three months administration. **(I)** H&E staining images of aorta in 200x microscopes, n = 5. **(J)** H&E staining images of aorta in 400× microscopes, n = 5. **(K)** Oil red O staining images of aorta, n = 7–9. Data are the mean ± SD. *p < 0.05, ***p < 0.001 vs. AS group.

The body weight of mice in different groups was measured and compared. As shown in **Figures 1A, B**, both low-dose and high-dose alisol A effectively reduced the weight gain of *ApoE^−/−^* mice fed with high-fat diet. Of note, alisol A did not affect food intake in these mice. Interestingly, administration of atorvastatin did not decrease the body weight in the high-fat diet mice. These results indicated that Alisol A has a profound effect on mice energy balance and metabolism.

### Plasma Lipoproteins Analysis

There were no statistically significant differences in plasma lipidlevels between treatment groups (data not shown).

### Alisol A Promoted Plaque Regression

To assess the effect of alisol A on AS, we monitored the progress of atherosclerotic plaques formation in the tested mice (**Figures 1C–E**). At 4, 8, and 12 weeks after drug administration, the high-resolution small animal ultrasound system was used to monitor the area and diameter of plaques in the aortic arch of mice. As shown in **Figures 1F–H**, while the plaque area in aortic arch of mice in the AS group increased, plaque in AS+A High group decreased markedly. There is a statistically significant difference between AS+A High group and the AS group in one and two months after drug administration. In addition, the diameter of aortic arch in mice in AS group became narrower, whereas that in AS+A High group became wider. There is a statistically significant difference between AS+A High group and the AS group in two months after drug administration, but the difference is not significant between AS+AT or AS+A Low group and the AS group. At 12th week, the aorta of the tested mice was taken for oil red O and H&E staining. Arterial plaques in the high-dose A treated mice were significant reduced compared with that of the AS group (**Figures 1I–K**). These results demonstrated that alisol A significantly slowed down the process of AS induced by high-fat diet in *ApoE^−/−^* mice, reduced the aortic plaque area of the mice, and widened the inner diameter of aortic arch.

### Alisol A Reduced the Expression of Cell Adhesion Molecules and Inflammatory Factors

**Figure 2:**
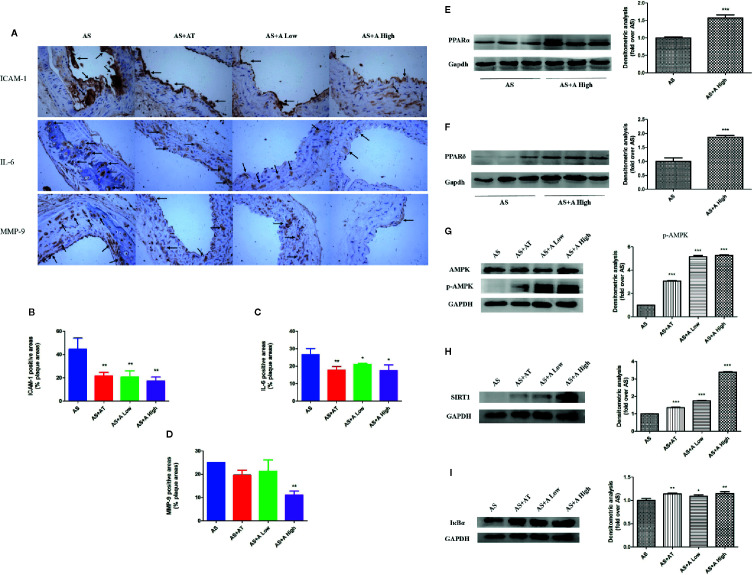
Alisol A reduces cell adhesion molecules and inflammatory factors in blood vessels and activate PPARα/δ in liver tissue. **(A)** Immunohistochemistry on ICAM-1, IL-6, and MMP-9; Magnification, 200×. **(B)** Quantitative analysis of the ICAM-1 positive area. **(C)** Quantitative analysis of the IL-6 positive area. **(D)** Quantitative analysis of the MMP-9 positive area. **(E)** Protein levels of PPARα in ApoE*^−/−^* mice liver tissue. **(F)** Protein levels of PPARδ in ApoE^−/−^ mice liver tissue. **(G)** Protein levels of p-AMPK and AMPK in ApoE^−/−^ mice liver tissue. **(H)** Protein levels of SIRT1 in ApoE−/− mice liver tissue. **(I)** Protein levels of IκBα in ApoE^−/−^ mice liver tissue. Data are the mean ± SD. *p < 0.05; **p < 0.01; ***p < 0.001 vs. AS group.

We examined the expressions of inflammatory factors interleukin 6 (IL-6), intercellular adhesion factor (ICAM-1), and metalloproteinase 9 (MMP-9) in the aortic plaque of mice in each group by immunohistochemistry (**Figure 2A**). It was found that administration of atorvastatin and low-dose alisol A significantly inhibited the expression of ICAM-1 and IL-6 compared with that in the AS group. Administration of high-dose alisol A significantly inhibited the expression of ICAM-1 (**Figure 2B**), IL-6 (**Figure 2C**), and MMP-9 (**Figure 2D**), which indicated that alisol A suppresses the inflammatory reactions in aortic plaque.

### Alisol A Activated PPARα, PPARδ Expression, and AMPK/SIRT1 Signaling Pathway in Mouse Liver

In order to explore the mechanisms of action of alisol A on AS, we evaluated the expression of PPARα, PPARδ, and AMPK/SIRT1 pathway in the liver of *ApoE^−/−^* mice by immunoblotting. As shown in **Figures 2E, F**, administration of alisol A significantly increased the expression of PPARα and PPARδ when compared with that from the AS group (**Figures 2E, F**). As shown in **Figures 2G–I**, administration of alisol A significantly increased the expression of p-AMPK, SIRT1, and IκBα in the liver of *ApoE^−/−^* mice. All these data suggested that alisol A activats AMPK/SIRT1 pathway to regulate the AS in *ApoE^−/−^* mice.

### Alisol A Did Not Affect Cell Viability

**Figure 3:**
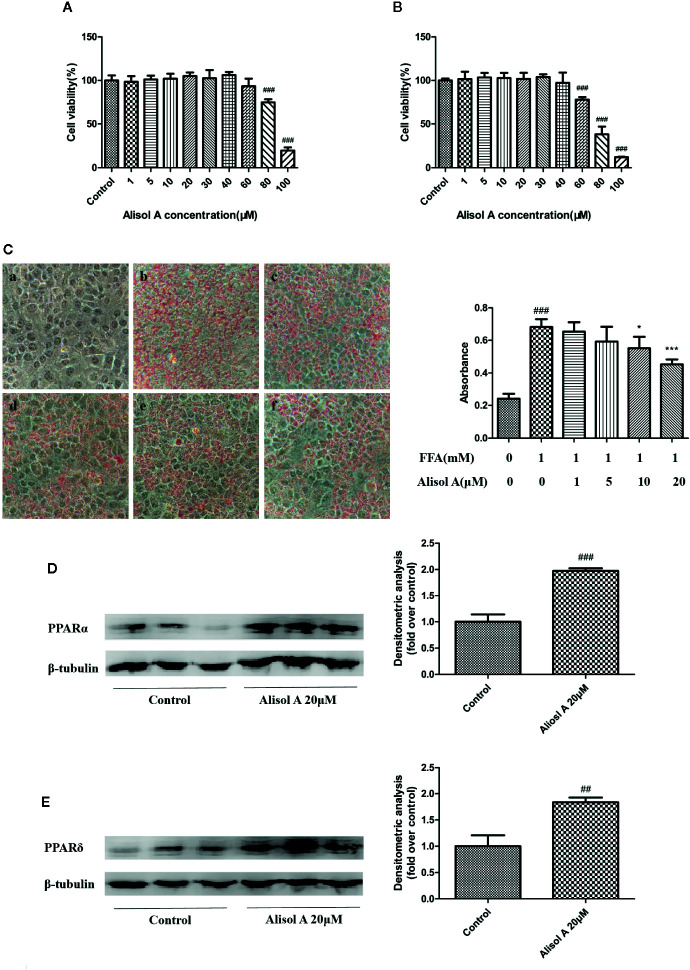
Alisol A reduces lipid accumulation in HepG2 cells. **(A)** Alisol A on cell viability of HepG2 cells treated for 24 h. **(B)** Alisol A on cell viability of HepG2 cells treated for 48h. **(C)** Oil Red O staining revealed lipid accumulation in HepG2 cells treated by different concentration of alisol A in 400x microscopes (a, Contorl; b, FFA; c, FFA+alisol A 1μM; d, FFA+alisol A 5μM; e, FFA+alisol A 10μM; f, FFA+alisol A 20μM), n = 4. **(D)** Protein levels of PPARα in HepG2 cells. **(E)** Protein levels of PPARδ in HepG2 cells. Data are the mean ± SD. ^##^p < 0.01; ^###^p < 0.001 vs. control group; *p < 0.05; ***p < 0.001 vs. model group.

We examined the effect of alisol A on the viability of HepG2 cells. As shown in **Figures 3A, B**, exposed to 0–30 μM alisol A for 0–48 h did not affect the viability of HepG2 cells as measured by the CCK assay.

### Alisol A Inhibited FFA-Induced Lipid Accumulation in HepG2 Cells

Oil Red O staining was used to evaluate the effect of alisol A on lipid accumulation in HepG2 cells. FFA (1mM, OA: PA = 2: 1) and indicated concentrations of alisol A were used to treat HepG2 cells for 24 h. As shown in **Figure 3C**, FFA induced the accumulation of a large number of lipid in HepG2 cells. Compared with that in the FFA group, lipid accumulation in alisol A–treated HepG2 cells was dose-dependent, and the group of 20 μM was significantly lower than that of the model group (*p* <0.001).

### Alisol Activated PPARα and PPARδ Expression in HepG2 Cells

We also examined the effects of alisol A on PPARα and PPARδ in HepG2 cells. Consistent with the finding in liver tissue from the tested mice, the levels of PPARα and PPARδ in HepG2 cells were markedly upregulated in alisol A–treated HepG2 cells (**Figures 3D, E**).

### Alisol A Reduced the mRNA Expression of Fatty Acid Synthase (FAS)

**Figure 4:**
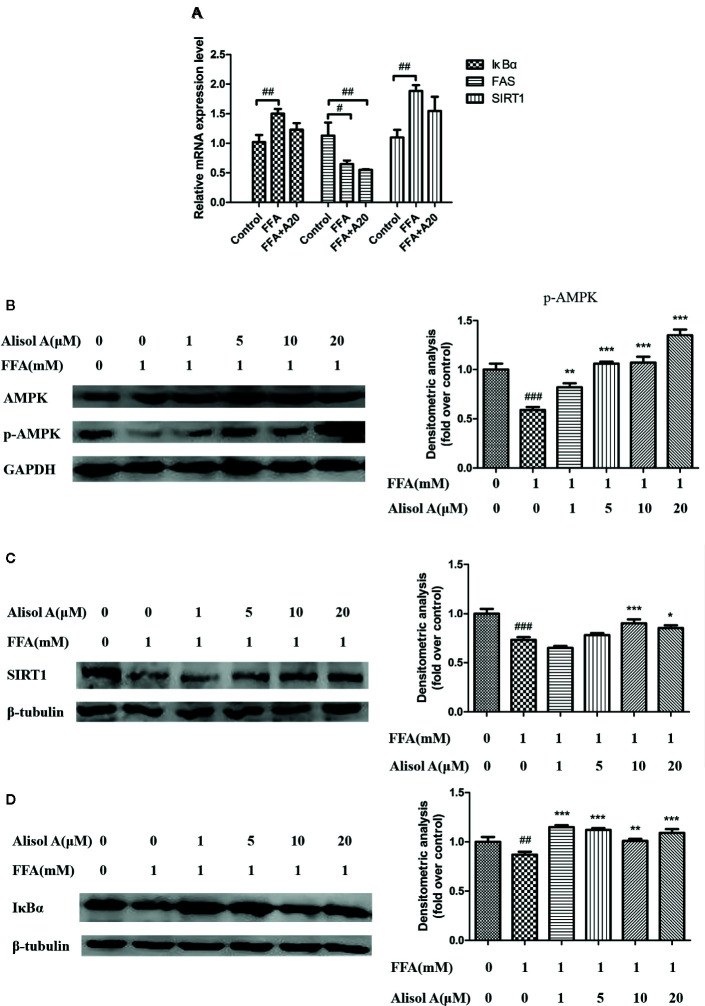
Alisol A activates AMPK/SIRT1 signaling pathway and IκBα in HepG2 cells. **(A)** Relatively mRNA expression levels of FAS, SIRT1, and IκBα, n = 3. **(B)** Protein levels of p-AMPK and AMPK in HepG2 cells, n = 3. **(C)** Protein levels of SIRT1 in HepG2 cells, n = 3. **(D)** Protein levels of IκBα in HepG2 cells, n = 3. Data are the mean ± SD. ^#^p < 0.05; ^##^p < 0.01; ^###^p < 0.001 vs. control group; *p < 0.05; **p < 0.01; ***p < 0.001 vs. FFA group.

We tested mRNA expression levels of Fatty acid synthase(*FAS*), *SIRT1*, and *IκBα* by and quantitative real-time PCR. The results suggested that alisol A could reduce the mRNA expression levels of *FAS*. But there was no significant effect on the mRNA expression levels of *SIRT1* and *IκBα* between FFA group and FFA+alisol A group (**Figure 4A**).

### Alisol A Stimulated the AMPK/SIRT1 Signaling Pathway in HepG2 Cell

To further understand the mechanisms of the effects of alisol A on AS, we analyzed the AMPK/SIRT1 signaling pathway in HepG2 cells exposed to different concentrations of alisol A. Immunoblotting analyses showed that p-AMPK and SIRT1 were significantly down-regulated in HepG2 cells treated with FFA, whereas the overall level of AMPK was not affected (**Figure 4B**). Interestingly, alisol A abolished the down-regulation of p-AMPK and SIRT1 expression in HepG2 cells induced by FFA. As shown in **Figures 4B, C**, this effect of alisol A was concentration-dependent. Furthermore, FFA markedly reduced the expression of IκBα that blocks the activation of proinflammatory NF-κB. Alisol A effectively prevented FAA-induced IκBα down-regulation (1 and 5 μM) (**Figure 4D**), which may contribute to its anti-inflammatory action in the plaques.

## Discussion

We have previously showed that the extract of AR has hypolipemic effect in various animal models such as golden hamsters, New Zealand rabbits and SD rats, suggesting that it could be used as a new hypolipidemic drug in clinical trials (Zhihong, 2017). As alisol A is the main component of AR extract, we assessed its effect on plasma lipid, aorta diameter and plaque formation in apoE-deficient mice fed with high-fat diet. ApoE is an efficient ligand for the interaction of lipoproteins with lipoprotein receptors; therefore, it plays a central role in the metabolic fate of plasma lipoproteins and cholesterol (Rall and Mahley, 1992). AopE gene deletion can cause various abnormalities in lipid metabolism (Walden and Hegele, 1994). We suspected that the above results may be caused by abnormal lipid metabolism, which also suggested that alisol A may exert anti-atherosclerotic effect by lowering plasma lipids. The non-invasive high-resolution small animal ultrasound system makes it possible to perform quantitative assessment in live mice, although the imaging plaque could be affected by the equipment and operator’s experience (Zhang et al., 2015). The results of non-invasive small animal ultrasound examination in the mice three months after the drug administration demonstrated no statistical difference among the groups. However, the plaque of AS+A High group in three months was significantly reduced compared with the results before alisol A administration. Both AT and AS+A Low group showed a tendency of decreasing plaque. The inner diameter of aortic arch showed a tendency of increasing after three months in each group compared with those before alisol A administration. The results of Oil Red O staining showed that it significantly reduced the aortic plaque area of the tested mice in AS+A High group. Aortic plaque in the AT group also showed a decreasing trend, but it did not reach a statistical difference, which could be due to the small sample size. The reason for the inconsistency of the two test results may also be that, the non-invasive small animal ultrasound system only tests the aortic arch, but Oil Red O staining detects the whole aorta.

It has been demonstrated that PPARα is a key nuclear receptor of liver cells, whose activation reduces the levels of triglyceride and VLDL and increases HDL level in plasma (Mirza et al., 2019). PPARα agonists inhibit the formation of AS by suppressing the formation of foam cells through the ABCA1 pathway (Li et al., 2004). PPARδ is an unsaturated fatty acid receptor, expresses in most cells including skeletal muscle and macrophages, and acts as a critical regulator of cellular energy expenditure. Its activation can suppress inflammation, reduce lipase activity, and inhibit low density lipoprotein production (Liu et al., 2018). It has been reported that PPARδ regulated fatty acid transport, β-oxidation, and mitochondrial respiration (Wang et al., 2003). Furthermore, PPARδ agonist suppresses atherosclerotic lesion progression by improving the serum lipoprotein profiles (Naya et al., 2016). Our data indicated that alisol A is a dual agonist for PPARα/δ. It is conceivable that increased expression of PPARα and PPARδ expression contributes to its anti-atherosclerotic effect.

Additionally, expression of inflammatory factors also plays an important role in the progression of AS (Huber et al., 1999; Kim and Ko, 2014; Raggi et al., 2018). In this study, we used immunohistochemistry to assess the expression of inflammatory factors IL-6, ICAM-1, and MMP-9 in the aortic plaque site of mice in each group. The results demonstrated that high-dose alisol A significantly inhibited the expression of the ICAM-1, IL-6, and MMP-9. It remains to be explored whether these actions of alisol A are mediated by activation of PPARα and PPARδ.

It was reported that alisol A can activate the phosphorylation of AMPK (Ho et al., 2019). AMPK reduces lipid accumulation by regulating the expression of PPARα (Chen et al., 2013; Kang et al., 2015), whereas histone deacetylase SIRT1 senses the level of energy and helps cells to resist external stress and improve metabolism (Stein and Matter, 2011). The activation of the AMPK/SIRT1 pathway can inhibit lipid accumulation and improve intracellular lipid metabolism (Zhang et al., 2019). In this study, we found that FAA induced dyslipidosis in HepG2 cells, which was associated with reduced levels of p-AMPK and SIRT1, whereas alisol A significantly up-regulated p-AMPK and SIRT1 in HepG2 cells. Interestingly, alisol A prevents the reduction of IκBα, which may account for the finding that activating AMPK/SIRT1 pathway could reduce NF-κB activity (Abedimanesh et al., 2020).

Taken together, our study indicates that alisol A is a multi-targeted anti-atherosclerotic drug, which activates AMPK/SIRT1 pathway and PPARα/δ to improve intracellular lipid metabolism. Activation of AMPK/SIRT1 pathway prevents the reduction of IκBα, which inhibits the activation of proinflammatory transcription factor NF-κB. Thus, the ability of alisol A to regulate lipid metabolism and suppress inflammation makes it a promising lead compound for developing novel and effective anti-AS drugs.

## Data Availability Statement

Data will be available upon request.

## Ethics Statement

The animal study was reviewed and approved by Laboratory animal management, Welfare and Ethics Committee of Shanghai Institute of Pharmaceutical Industry.

## Author Contributions

ZHC conceived and designed the study. KW, BZ, and DS performed the experiments. KW wrote the paper and accomplished cell part. BZ and DS accomplished animal part. JX, WH, JY, CG, and ZBC helped animal part. All authors contributed to the article and approved the submitted version.

## Funding

Shanghai “science and technology innovation action plan” biomedical science and technology support project no. 12401901300.

## Conflict of Interest

The authors declare that the research was conducted in the absence of any commercial or financial relationships that could be construed as a potential conflict of interest.
